# Isolation and characterization of five novel probiotic strains from Korean infant and children faeces

**DOI:** 10.1371/journal.pone.0223913

**Published:** 2019-10-31

**Authors:** Sun-Young Kook, Eui-Chun Chung, Yaelim Lee, Dong Wan Lee, Seokjin Kim

**Affiliations:** R&D Institute, BioEleven Co., Seoul, Republic of Korea; National Dairy Research Institute, INDIA

## Abstract

Probiotics are dietary supplements containing viable, non-pathogenic microorganisms that interact with the gastrointestinal microflora and directly with the immune system. The possible health effects of probiotics include modulating the immune system and exerting antibacterial, anticancer, and anti-mutagenic effects. The purpose of this study was to isolate, identify, and characterize novel strains of probiotics from the faeces of Korean infants. Various assays were conducted to determine the physiological features of candidate probiotic isolates, including Gram staining, 16S rRNA gene sequencing, tolerance assays to stimulated gastric juice and bile salts, adherence ability assays, antibiotic susceptibility testing, and assays of immunomodulatory effects. Based on these morphological and biochemical characteristics, five potential probiotic isolates (*Enterococcus faecalis* BioE EF71, *Lactobacillus fermentum* BioE LF11, *Lactobacillus plantarum* BioE LPL59, *Lactobacillus paracasei* BioE LP08, and *Streptococcus thermophilus* BioE ST107) were selected. *E*. *faecalis* BioE EF71 and *L*. *plantarum* BioE LPL59 showed high tolerance to stimulated gastric juice and bile salts, and *S*. *thermophilus* BioE ST107 as well as these two strains exhibited stronger adherence ability than reference strain *Lactobacillus rhamnosus* GG. All five strains inhibited secretion of lipopolysaccharide-induced pro-inflammatory cytokines IL-6 and TNF-α in RAW264.7 macrophages *in vitro*. *L*. *fermentum* BioE LF11, *L*. *plantarum* BioE LPL59, and *S*. *thermophilus* BioE ST107 enhanced the production of anti-inflammatory cytokine IL-10. Overall, our findings demonstrate that the five novel strains have potential as safe probiotics and encouraged varying degrees of immunomodulatory effects.

## Introduction

Probiotics defined as “living micro-organisms, which upon ingestion in certain numbers, exert health benefits beyond inherent basic nutrition”, have become a major topic of lactic acid bacteria research over the past 20 years [1]. Probiotics are usually considered dietary supplements and contain viable, non-pathogenic microorganisms that interact with the gastrointestinal microflora and directly with the immune system [2]. Possible health effects of probiotics include modulating the immune system; antibacterial, anticancer, and anti-mutagenic activities; and preventing cancer recurrence [3–6]. Certain members of the *Lactobacillus*, *Bifidobacterium*, *Streptococcus*, and *Enterococcus* genera are thought to be beneficial for human health when ingested and are reported to exert anti-inflammatory properties [7]. Members of these genera have been shown to be useful in the treatment and prevention of immune and intestinal disorders, including allergic diseases, diarrhoea, and chronic inflammatory diseases [8–10]. However, these beneficial effects have been associated with a minority of strains, and other strains and same species cannot be assumed to have the same effects [11]. The effects of probiotics on immune-modulatory cytokine level have been shown to be highly diverse and strain-dependent as well as cell type-specific.

For probiotics to be successful, a strain should be able to colonize the gastrointestinal tract and promote host health through its metabolic activities. The safety and functional properties of the strains, such as antibiotic resistance and adherence to intestinal mucosa cells, and the possibility of immunomodulation are very important for the selection of potential probiotic strains, they should be studied using reliable *in vitro* screening methods [12].

The modulation of immune responses, such as the suppression of inflammation, is a major part of the crosstalk between bacteria and epithelial cells. Previous studies have reported that some bacteria induce the secretion of pro-inflammatory cytokines, such as tumour necrosis factor (TNF)-α and interleukin (IL)-6, whereas others promote the secretion of anti-inflammatory cytokines such as IL-10 [13–16]. These cytokines contribute to defence mechanisms that participate in host immunity in response to external invasion, but they may induce immune-pathological disorders when secreted in excess. Macrophages derived from monocytes play a central role in initiating the primary defence system of host immunity and can be activated by microbial components such as endotoxins, lipopolysaccharides (LPS), and lipoteichoic acids (LTA) [17]. This enables the recognition of foreign objects that trigger a cascade of immunological defence mechanisms, such as the production of pro- and anti-inflammatory cytokines [18].

In this study, in order to isolate, identify, and characterize novel strains of probiotics, 20 strains were isolated from Korean infant stool samples and were examined for their acid and bile tolerance, adherence to intestinal mucus, and effects on the induction of known pro-inflammatory and anti-inflammatory cytokines in LPS-stimulated macrophages.

## Materials and methods

### Subjects and ethics statement

As they age, the number of beneficial bacteria in the intestinal environments is generally lower, which can result in an imbalance in bacterial community composition. However, the beneficial bacteria including *Lactobacillus* and *Bifidobacterium* spp. are abundant in infants [19, 20]. The study included faecal samples from 10 healthy infants aged from 1 to 47 months, with no exclusions based on delivery or feeding mode. Mothers and infants were in good health (self-reported). Subjects were excluded if the infant had a gastrointestinal disorder or had taken antibiotics in the previous 14 days, if the infant had been ill in the previous 7 days, or if the infant was administered oral probiotics. This study was approved by the Public Investigational Review Board designated by the Ministry of Health and Welfare (IRB number: P01-201712-33-002). Written informed consent was obtained from all parents according to the institutional guidelines. Fresh faecal samples were collected by the participants and immediately stored in home freezers until delivery to the experimental laboratory within 24 h of sample collection. Samples were placed in labelled collection tubes and stored at -80°C until analysis.

### Faecal samples and bacterial growth conditions

Fresh faecal samples were mixed with de Man, Rogosa and Sharpe (MRS; Difco, Detroit, MI, USA) broth at a ratio of 1:9 and homogenized using a BenchMixer (Benchmark, Sayreville, NJ, USA) according to the method of Chung *et al* [21]. Homogenized samples were incubated under anaerobic conditions at 37°C for 3 h. Each sample was streaked onto MRS, blood liver (BL; MB Cell, Los Angeles, CA, USA), and bismuth sulphite (BS; Difco) agar, which is selective for probiotics. The plates were incubated at 37°C for 48–72 h under anaerobic conditions. For each faecal culture sample, 118 colonies were randomly selected and purified on MRS broth medium (Difco) for identification. Isolates were stored frozen at -80°C in glycerol until subsequent analyses.

### Isolation and identification of lactic acid bacteria

For identification, the cell morphology of selected isolates was examined by microscopy. Selected isolates were identified by Gram staining, Analytical profile index (API) 50 CHL kit (BioMerieux, Marcy l’Etoile, France) or API 20E Strep kit (BioMerieux), and 16S ribosomal (r)RNA gene sequencing. The 16S rRNA gene was amplified by direct PCR using the following universal primers: forward, 5ʹ-GAGTTGGATCCTGGCTCAG-3ʹ and reverse, 5ʹ-AAGGAGGGGATCCAGCC-3ʹ. DNA was extracted from each strain with a commercial G-spin kit for bacterial genomic DNA extraction (Intron, Korea) according to the manufacturer’s instructions. Sequencing of the 16S rRNA gene was performed by a commercial sequencing facility (Macrogen, Seoul, Korea) with an ABI 3730XL DNA analyzer. The 16S rDNA sequences were analysed using the GenBank (NCBI, Bethesda, MD, USA) database, and identification was performed on the basis of 16S rDNA sequence homology using the BLAST database. CLUSTAL X [22] was used to construct multiple alignments of 16S rRNA gene sequences. A phylogenetic tree was constructed in MEGA version 7 [23] using the neighbor-joining method [24]. Bootstrap analysis was based on 1,000 neighbor-joining datasets [25].

### Tolerance to low pH and bile salts

For determining the tolerance of the isolated strains to low pH and bile salts, an *in vitro* methodology was used [26], which mimics conditions encountered during *in vivo* human upper gastrointestinal transit. Tolerance was examined by monitoring bacterial growth. In brief, artificial gastric juice was prepared by suspending pepsin (Sigma-Aldrich, St. Louis, MO, USA) in MRS broth to a final concentration of 250 units/ml and adjusting the pH to 2.5 with 1 N HCl using a Model S220-K pH meter (Mettler, Toledo, OH, USA). The strains were incubated at 37°C for 18 h and then centrifuged at 10,000 × *g* for 25 min at 4°C. After centrifugation, the supernatant was removed, and an equal volume of artificial gastric juice was added to the bacterial cell pellet and incubated at 37°C for 2 h. The pellets were collected by centrifugation at 10,000 × *g* for 25 min at 4°C and washed three times with phosphate-buffered saline (PBS). MRS broth containing 0.3% bile acids (Oxgall; Difco) was added to each pellet in artificial gastric juice and incubated at 37°C for 24 h. The number of bacterial colony-forming units (CFU) was determined on MRS agar plates. Assays were performed three times independently.

### *In vitro* adherence assay

The human intestinal epithelial cell line Caco-2 was acquired from the Korean Collection for Type Cultures (KCTC, Daejeon, Korea). For isolated strains with high resistance to low pH and bile salts, adherence to Caco-2 cell cultures was assessed. Caco-2 cells were cultured in modified Eagle’s medium (MEM; Corning, Corning, NY, USA) supplemented with 20% fetal bovine serum (Biowest, France), 100 U/ml penicillin, and 100 μg/ml streptomycin (Gibco, Waltham, MA, USA) at 37°C in 5% CO_2_. Caco-2 cells were seeded in a cell culture dish with a diameter of 60 mm (SPL Life Sciences, Gyeonggi, Korea) at 1.0 × 10^5^ cells/cm^2^ and cultured for 2 d. Before the adherence assay, Caco-2 cell monolayers were washed three times with PBS (Gibco), and the medium was replaced with antibiotic-free MEM. Each dish was inoculated with 100 μl (10^8^ bacteria) of 18 h culture and incubated for 2 h at 37°C in 5% CO_2_. After 2 h of incubation, the monolayers were washed three times with PBS, and 200 μl of 1% Triton-X 100 (Sigma-Aldrich) was added. An aliquot of 1 ml of the homogenate was added to an MRS agar plate by serial dilution. The percentage of bacteria that adhered to the plate was then calculated. All experiments were performed three times independently. The commercial strain *Lactobacillus rhamnosus* GG (*L*.*GG*) from the American Type Culture Collection (Manassas, VA, USA; ATCC 53103) was grown and used as a control for this assay.

### Antibiotic resistance profiles of isolates

The antibiotic resistance profiles of selected isolates were determined using disc diffusion assays with 13 discs purchased from Bio-Rad (Hercules, CA, USA) containing the following antibiotics: vancomycin (VAN, 30 μg), erythromycin (ERY, 15 μg), tetracycline (TET, 30 μg), gentamycin (GMN, 10 μg), chloramphenicol (CHL, 30 μg), ampicillin (AMP, 10 μg), streptomycin (SMN, 10 μg), ciprofloxacin (CIP, 5 μg), rifampin (RIF, 5 μg), imipenem (IPM, 10 μg), trimethoprim (TMP, 5 μg), clindamycin (CMN, 2 μg), and kanamycin (KMN, 30 μg). A volume of 100 μl of an overnight culture suspension of each isolate (equivalent to 10^8^ bacteria/ml) was spread on an MRS agar (Difco) plate. *E*. *coli* KCTC1682, used as a control, was spread on Müller-Hinton agar (MHA) plates (Difco). Discs with antibiotics were placed onto the solidified MHA or MRS agar with sterile tweezers for 15 min. Three discs were placed in each dish with a distance of more than 24 mm between the centres of the discs and more than 15 mm between the edge of each disc and the inner edge of the dish. Plates were incubated at 37°C for 24 h under anaerobic conditions, except for *E*. *coli* KCTC1682, which was grown under aerobic conditions. After 24 h, the diameter of the inhibition zone around each antibiotic disc was measured with a SCAN-500 (Interscience, France) and compared with a known standard provided by the Clinical and Laboratory Standards Institute (CLSI) guidelines. Breakpoints were calculated as previously described [27].

### Immunomodulatory cytokine analysis

RAW264.7 murine macrophages were acquired from the KCTC and were maintained in Dulbecco’s MEM (DMEM; Corning) supplemented with 20% fetal bovine serum (Biowest), 100 U/ml penicillin, and 100 μg/ml streptomycin (Gibco) at 37°C in 5% CO_2_. RAW264.7 cells were seeded in a 24-well plate (SPL) at 1 × 10^6^ cells/cm^2^ and cultured for 4 h at 37°C in a 5% CO_2_ incubator. The levels of cytokines produced following stimulation with each bacterial isolate were compared with those observed in RAW264.7 cells in DMEM alone as a negative control and in cells cultured with LPS (1 μg/ml; Sigma-Aldrich) as a positive control. Before treatment of RAW264.7 cells with bacteria, RAW264.7 cell monolayers were washed three times with PBS (Gibco), and the medium was replaced with antibiotic-free MEM. After culture in MRS broth for 18 h at 37°C in an anaerobic incubator, bacterial cells (1 × 10^8^ CFU/ml) were washed with PBS and added to culture plates containing RAW264.7 cells. All cells except the negative controls were stimulated with LPS (1 μg/ml) for 24 h. After stimulation, culture supernatants were collected and centrifuged at 13,000 rpm for 3 min and stored at -20°C until cytokine analysis [28]. Cytokine levels (IL-6, TNF-α, and IL-10) were measured using commercial ELISA kits (Cusabio Biotech, Wuhan, China).

### Statistical analysis

All data are expressed as the mean ± SEM. Statistical analysis was performed with GraphPad Prism 5 (San Diego, CA, USA). Differences in abundances of bacterial species between the mean values for different treatments with the isolated strains or their supernatants were analysed by one-way ANOVA with turkey as appropriate. (*p < 0.05, **p < 0.01, and ***p < 0.001).

## Results

### Isolation and identification of bacteria

A total of 10 faecal samples from infants under 47 months of age were obtained from 10 healthy mothers regardless of their delivery mode. The faecal samples yielded 84 distinct bacterial isolates representing the genera *Bifidobacterium*, *Lactobacillus*, *Enterococcus*, *Klebsiella*, *Staphylococcus*, and *Streptococcus* (Table 1). Of these, we first selected 20 strains above 10^8^ CFU/ml and 20 isolates showed gram-positive and catalase negative reaction identified by gram-staining and API kit. We used the API 20 strep kit for *Enterococcus faecalis* and the API 50 CH fermentation system for the rest of the isolates to assess the carbohydrate fermentation ability of the isolated lactic acid bacterial (LAB) strains, and these results are shown in Table 2.

**Table 1:** Bacterial isolates by genus and species isolated from infant stool samples obtained from 10 mother’s infants (n = 10).

Bacterial genus and species	No. of isolates	Comments
*Bifidobacterium animalis* subsp. *lactis*	1	From 1 infant (ages 1 month)
*Bifidobacterium pseudocatenulatum*	8	From 2 infants (ages 1 and 47 months)
*Lactobacillus paracasei*	8	From 1 infant (ages 9 months)
*Lactobacillus plantarum*	43	From 3 infants (ages 0.5, 1, 11 months)
*Lactobacillus fermentum*	1	From 1 infant (ages 1 months)
*Enterococcus avium*	15	From 1 infant (ages 31 months)
*Enterococcus gallinarum*	3	From 2 infants (ages 24 and 31 months)
*Enterococcus malodoratus*	3	From 2 infants (ages 24 and 31 months)
*Enterococcus faecalis*	4	From 3 infants (ages 0.5, 1, 31 months)
*Enterococcus faecium*	27	From 3 infants (ages 7, 9, 24 months)
*Klebsiella pneumoniae*	3	From 1 infants (ages 7 months)
*Staphylococcus epidermidis*	1	From 1 infants (ages 0.5 months)
*Streptococcus thermophilus*	1	From 1 infant (ages 11 months)
Total number of unique isolates	118	From 10 infant (ages 0.5, 1, 7, 9, 11, 24, 31, 47 months)

**Table 2:** Identification of isolates using API 50 CH (*Lactobacillus fermentum*, *Lactobacillus paracasei*, *Lactobacillus plantarum*, *Streptococcus thermophilus*) and API 20 strep (*Eenterococcus faecalis*) kit.

	In agreement		
Strains^a^	Positive	Negative	% of agreement
*E*. *faecalis* BioE EF71	11	9	99.7
*L*. *fermentum* BioE LF11	12	38	81.2
*L*. *paracasei* BioE LP08	46	4	99.3
*L*. *plantarum* BioE LPL59	44	6	99.9
*S*. *thermophilus* BioE ST107	3	47	99.2

^**a**^All isolates were able to ferment glycerol, erythritol, D-Arabinose, L-Arabinose, L-Xylose, β Methyl-D-Xyloside, rhamnose, dulcitol, inositol, inulin, starch, glycogen, xylitol, D-Fucose, L-Fucose, D-Arabitol, L-Arabitol, 2 keto-gluconate, 5 keto-gluconate.

**Fig 1:**
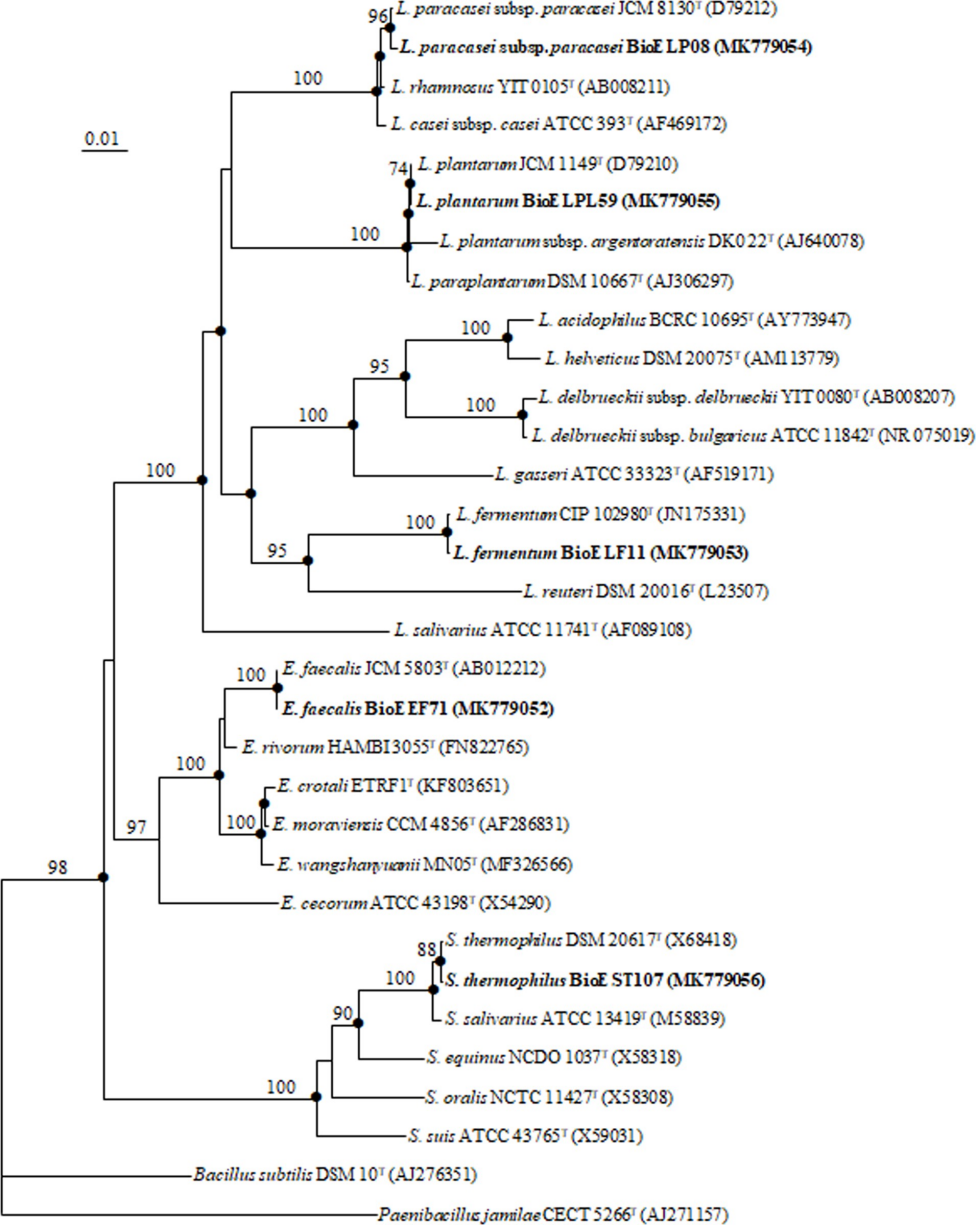
Phylogenetic tree showing the genetic relationships of the five isolates from Korean infant stools with the closest sequences identified in GenBank by BLAST. Neighbor-joining analyses were conducted with the Jukes and Cantor model using the PHYLIP package [68]. The sequence of *Paenibacillus jamilae* CECT 5266^T^ (AJ271157) served as the outgroup. Solid circles indicate that corresponding nodes were also recovered using maximum-likelihood [69] and maximum-parsimony [70] trees. Bootstrap values (expressed as percentages of 1,000 repetitions) >70% are indicated at each node. Bar, 0.01 substitutions per nucleotide position.

Next, the 20 selected strains were subjected to PCR and 16S rDNA sequencing and analysed using BLAST. Among the 20 isolates, five isolates exhibited >99.5% sequence identity to each of *E*. *faecalis*, *Lactobacillus fermentum*, *Lactobacillus paracasei*, *Lactobacillus plantarum*, and *Streptococcus thermophilus*. A phylogenetic tree was created to show the species relationships of the isolates (Fig 1). These five isolates were patent deposited in the Korean Collection for Type Cultures (KCTC) under the following strain names: *E*. *faecalis* BioE EF71 (KCTC 18627P), *L*. *fermentum* BioE LF11 (KCTC 18628P), *L*. *paracasei* BioE LP08 (KCTC 18629P), *L*. *plantarum* BioE LPL59 (KCTC 18630P), and *S*. *thermophilus* BioE ST107 (KCTC 18631P). The NCBI GenBank accession numbers of these sequences are MK779052, MK779053, MK7799054, MK7799055, and MK7799056, respectively.

### Acid and bile salt tolerance

Bacterial survival was assessed under conditions similar to those in the proximal part of the gastrointestinal tract at time intervals corresponding to the actual presence of lactobacilli in the intestines. The five isolates showed good survival in artificial gastric juice and in a solution containing 0.3% bovine bile salts. After 2 h of exposure to artificial gastric juice, the growth of *L*. *fermentum* BioE LF11 and *S*. *thermophilus* BioE ST107 was almost maintained at control levels, with the number of viable cells at 8.62 and 5.91 log CFU/ml, indicating survival rates of 99.4 and 73.2%, respectively. The growth of *E*. *faecalis* BioE EF71 and *L*. *plantarum* BioE LPL59 decreased somewhat, resulting in viability values of 4.82 and 4.39 log CFU/ml, respectively. In addition, after 24 h of exposure to bovine bile salt solution, the strong growth of *E*. *faecalis* BioE EF71, *L*. *paracasei* BioE LP08, *L*. *plantarum* BioE LPL59 resulted in viability values of 9.00, 8.80, and 8.99 log CFU/ml (Table 3). *E*. *faecalis* BioE EF71, *L*. *paracasei* BioE LP08, and *L*. *plantarum* BioE-LPL59 exhibited higher tolerances to acid and bile salts than *L*.*GG*. In addition, while *S*. *thermophilus* BioE ST107 exhibited slower growth than *L*.*GG* in terms of the total number of viable cells, *S*. *thermophilus* BioE ST107 exhibited increased survival after 24 h following bile acid treatment.

**Table 3:** Tolerance to acid and bile salts of isolates and control strain *Lactobacillus rhamnosus* GG.

Isolates	Log CFU/ml		
	0 h	2 h, pH 2.5	24 h, 0.3% oxgall
*E*. *faecalis* BioE EF71	9.14 ± 0.05	4.82 ±1.73	9.00 ± 0.02
*L*. *fermentum* BioE LF11	8.67 ± 0.02	8.62 ± 0.03	7.84 ± 0.01
*L*. *paracasei* BioE LP08	9.62 ± 0.01	6.52 ± 0.57	8.80 ± 0.04
*L*. *plantarum* BioE LPL59	9.69 ± 0.01	4.39 ± 0.09	8.99 ± 0.01
*S*. *thermophilus* BioE ST107	8.07 ± 0.04	5.91 ± 1.73	7.02 ± 0.04
*L*. *rhamnosus* GG	9.31 ± 0.03	9.51 ± 0.02	7.70 ± 0.02

### Adherence to intestinal cells

**Fig 2:**
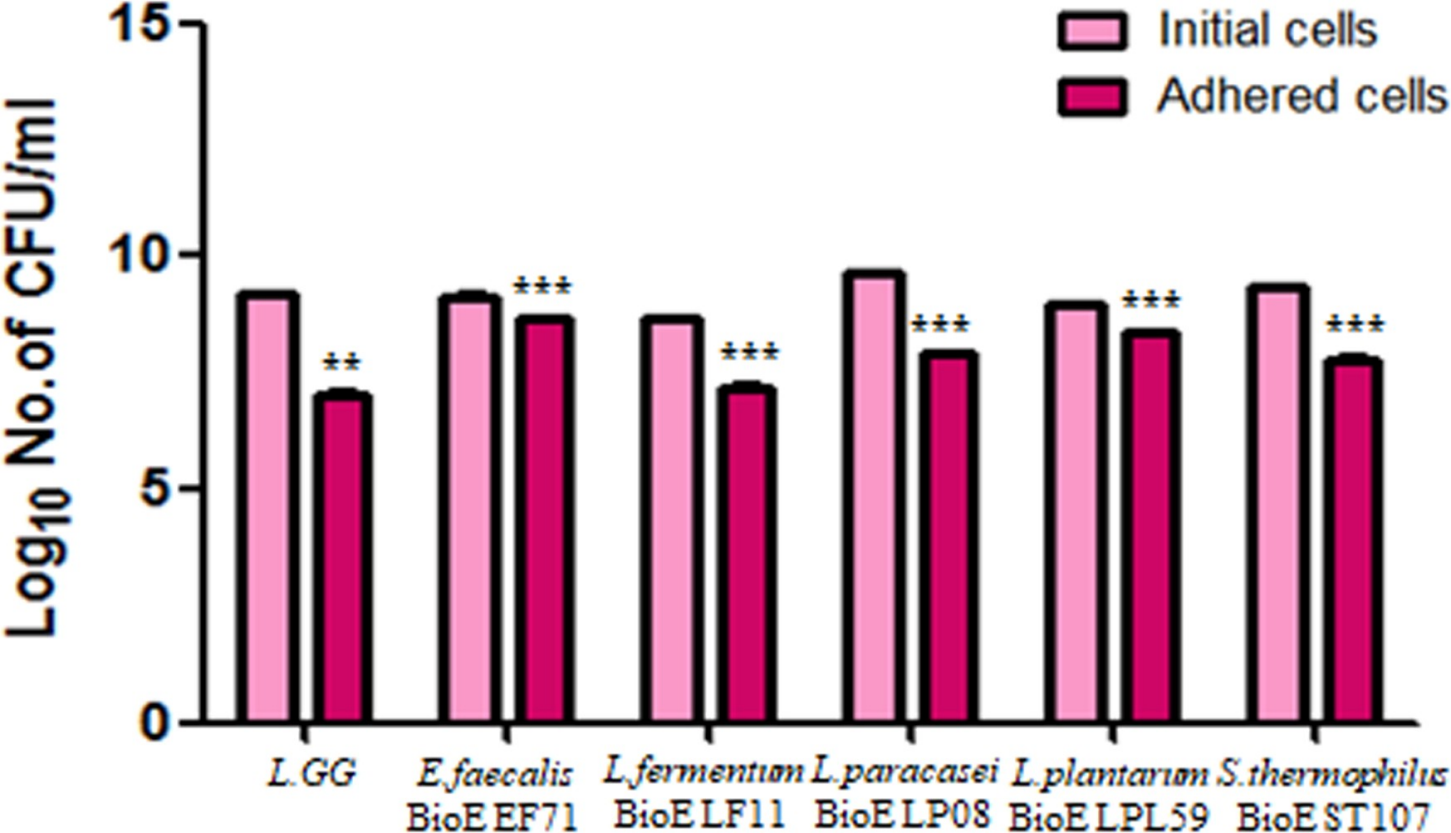
Adherence of *E*. *faecalis* BioE EF71, *L*. *fermentum* BioE LF11, *L*. *paracasei* BioE LP08, *L*. *plantarum* BioE LPL59, *S*. *thermophilus* BioE ST107, and *L*. *rhamnosus* GG to Caco-2 cells. After bacterial inoculation of Caco-2 monolayers, the number of bacteria that adhered to the plates was calculated. Pink numbers indicate the number of initial bacterial cells before addition to Caco-2 cells. Dark pink numbers indicate the mean number of adhered cells after addition to Caco-2 cells. Data shown are mean ± SEM of three independent experiments performed in triplicate. **p < 0.05, ***p < 0.001 versus initial cell number. Reference strain: *L*. *rhamnosus* GG. ***p < 0.001 versus *L*. *rhamnosus* GG.

To confirm the adherence of the five isolated strains to intestinal epithelial cells, we used the human intestinal epithelial cell line Caco-2. We determined the concentration (CFU/ml) of initial and adhered cells before and after adherence to Caco-2 cells, respectively. These values were 9.14 log CFU/ml and 7.79 log CFU/ml for *E*. *faecalis* BioE EF71 and 8.94 log CFU/ml and 8.4 log CFU/ml for *L*. *plantarum* BioE LPL59, indicating 30% survival rates for these two strains. Among the five strains, *E*. *faecalis*, *L*. *plantarum*, and *S*. *thermophilus* exhibited significantly improved adherence to Caco-2 cells compared to that of the reference strain (Figs 2 and S1).

### Antibiotic susceptibility

Disc diffusion assays were used to determine the antibiotic susceptibility profiles of the tested strains according to the antimicrobial drug sensitivity standards of the CLSI. The sensitivities of the five strains to 13 types of antibiotics are summarized in Table 4. The five strains were generally resistant to gentamicin (n = 4), kanamycin (n = 5), trimethoprim (n = 5), and vancomycin (n = 4) and were mostly sensitive to ampicillin (n = 4), chloramphenicol (n = 5), imipenem (n = 5), and rifampicin (n = 3). The resistance rates of the three species of *Lactobacillus* ranged from 38.5 to 53.8%, and these three isolates had similar resistance patterns. All three *Lactobacillus* strains, as well as the *L*.*GG* reference strain, were resistant to vancomycin. The resistance rate of *S*. *thermophilus*, which showed the highest antibiotic susceptibility, was 30.8%.

**Table 4:** Characteristics and antibiotic resistance profiles of isolates and *Eeschericia coli*.

Antibiotics^a^	Conc.	Diameter (mm) of inhibition zone						
		*E*. *coli*	*E*. *faecalis* BioE EF71	*L*. *fermentum* BioE LF11	*L*. *paracasei* BioE LP08	*L*. *plantarum* BioE LPL59	*S*. *thermophiles* BioE ST107	*L*. *rhamnosus GG*
AMP	30 ㎍	19.97 ± 0.15	33.97 ± 1.92	23.00 ± 1.04	31.82 ± 0.15	33.90 ± 0.82	31.8 ± 2.52	22.40 ± 1.15
CIP	5 ㎍	32.13 ± 2.84	18.90 ± 0.78	ND	16.07 ± 0.59	ND	14.23 ± 1.17	16.00 ± 1.1
CHL	30 ㎍	24.30 ± 2.00	28.10 ± 2.57	23.20 ± 1.11	26.71 ± 0.46	23.93 ± 2.05	28.70 ± 1.56	25.70 ± 0.10
CMN	2 ㎍	ND	ND	21.57 ± 1.67	36.02 ± 0.41	15.77 ± 0.21	27.63 ± 1.91	21.83 ± 1.58
ERY	15 ㎍	ND	ND	22.87 ± 3.10	31.21 ± 0.58	23.13 ± 0.21	28.23 ± 1.19	27.80 ± 0.36
GMN	10 ㎍	17.30 ± 0.26	ND	8.83 ± 0.06	10.18 ± 0.66	8.83 ± 0.06	12.00 ± 2.36	8.73 ± 0.12
IPM	10 ㎍	27.73 ± 0.55	29.80 ± 0.96	36.17 ± 1.16	27.54 ± 0.65	38.53 ± 1.96	33.80 ± 0.17	24.47 ± 1.32
KMN	30 ㎍	16.63 ± 0.75	ND	8.80 ± 0.0	ND	ND	8.03 ± 0.75	ND
RIF	5 ㎍	13.00 ± 0.30	17.50 ± 0.69	24.33 ± 0.83	28.07 ± 0.52	17.43 ± 0.51	23.13 ± 0.80	27.43 ± 1.46
SMN	10 ㎍	13.63 ± 0.50	ND	ND	ND	10.37 ± 1.34	12.70 ± 0.56	ND
TET	30 ㎍	23.37 ± 0.29	11.10 ± 0.36	22.13 ± 1.10	31.55 ± 0.27	17.83 ± 1.11	29.63 ± 2.68	29.00 ± 1.47
TMP	5 ㎍	18.90 ± 1.54	ND	7.83 ± 0.64	ND	4.03 ± 6.99	ND	ND
VAN	30 ㎍	ND	16.80 ± 0.26	ND	ND	ND	18.03 ± 0.57	ND

^a^AMP, ampicillin; CIP, ciprofloxacin; CHL, chloramphenicol; CMN, clindamycin; ERY, erythromycin; GMN, gentamicin; IPM, imipenem; KMN, kanamycin; RIF, rifampicin; SMN, streptomycin; TET, tetracycline; TMP, trimethoprim; VAN, vancomycin. *Note*. Diameter of the disc is 6 mm.

### Immunomodulatory effects

**Fig 3:**
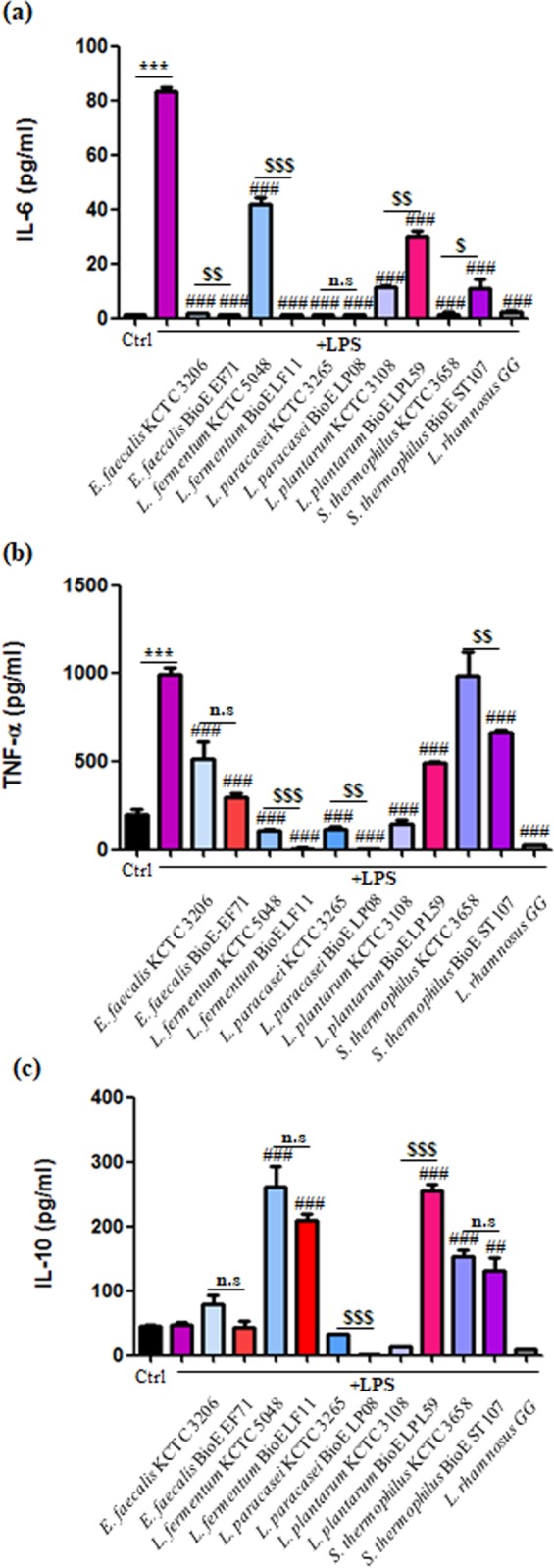
Effects of *E*. *faecalis* BioE EF71, *L*. *fermentum* BioE LF11, *L*. *paracasei* BioE LP08, *L*. *plantarum* BioE LPL59, *S*. *thermophilus* BioE ST107, and the reference strain *L*. *rhamnosus* GG on the secretion of IL-6 (a), TNF-α (b), and IL-10 (c) in LPS-stimulated RAW264.7 cells. Bacterial cells were added to RAW264.7 cells, followed by stimulation with LPS (except in negative control). Cytokine levels were measured by commercial ELISA kits. ***p < 0.001 versus negative control; ^##^*p* < 0.01, ^###^*p* < 0.001 versus LPS-treated sample; ^$ $^*p* < 0.01, ^$ $ $^*p* < 0.001 versus KCTC registered strain of the same species.

Murine macrophage RAW264.7 cells were stimulated with the five isolated strains, and the concentrations of IL-6, TNF-α, and IL-10 in the culture supernatants of the RAW264.7 cells were measured using ELISA kits. The IL-6 concentration in RAW264.7 cells cultured with LPS (1 μg/ml) was 83.1 ± 3.42 pg/ml, while minimal IL-6 levels were detected in untreated RAW264.7 cells (Figs 3a and S2 a). The LPS-induced increase in IL-6 levels was attenuated in cells stimulated with *E*. *faecalis* BioE EF71, *L*. *fermentum* BioE LF11, *L*. *paracasei* BioE LP08, *L*. *plantarum* BioE LPL59, and *S*. *thermophilus* BioE ST107 (1.65 ± 0.39 pg/ml, 1.33 ± 0.2 pg/ml, 1.28 ± 0.19 pg/ml, 29.82 ± 3.84 pg/ml, and 11.03 ± 5.43 pg/ml, respectively). We also compared the secretion of each cytokine following stimulation with the five isolated strains to those observed with other strains previously reported to exhibit anti-inflammatory properties. Thus, *L*. *fermentum* BioE LF11 (1.33 ± 0.2 pg/ml) was more effective at attenuating IL-6 levels than *L*. *fermentum* KCTC 5048 (42 ± 4.42 pg/ml), and *E*. *faecalis* BioE EF71 (1.65 ± 0.39 pg/ml) was more effective than *E*. *faecalis* KCTC3206 (1.84 ± 0.07 pg/ml). The TNF-α concentration in RAW264.7 cells cultured with LPS (1 μg/ml) was 992.57 ± 64.23 pg/ml (Figs 3b and S2 b). TNF-α induction was attenuated by *E*. *faecalis* BioE EF71, L. *fermentum* BioE LF11, and *L*. *paracasei* BioE LP08 (296.27 ± 37.2 pg/ml, 8.73 ± 3.76 pg/ml, and 3.13 ± 3.13 pg/ml, respectively). TNF-α levels were more effectively reduced by *L*. *fermentum* BioE LF11 and *L*. *paracasei* BioE LP08 than by *L*. *fermentum* KCTC 5049 (111.18 ± 7.45 pg/ml) and *L*. *paracasei* KCTC 3265 (118.83 ± 27.77 pg/ml). For experiments with the anti-inflammatory cytokine IL-10, treatment with *L*. *fermentum* BioE LF11 (208.51 ± 19.01 pg/ml) and *L*. *plantarum* BioE LPL59 (254.61 ± 29.94 pg/ml) resulted in higher levels of IL-10 than the other strains (Figs 3c and S2 c).

### Discussion

The early microbial colonization of the gastrointestinal tract in infants has a major effect on health status and homeostasis [29]. Previous studies have shown that the direct effects of probiotics such as *Lactobacillus*, *Bifidobacterium*, *Streptococcus*, and *Enterococcus* include the upregulation of immunoglobulins such as IgA, downregulation of inflammatory cytokines, and enhancement of the gut barrier function [30]. However, because each strain differs in other characteristics, it is essential to select and identify probiotics with desired characteristics.

In the present study, we identified five probiotics isolates (*E*. *faecalis* BioE EF71, *L*. *fermentum* BioE LF11, *L*. *paracasei* BioE LP08, *L*. *plantarum* BioE LPL59, and *S*. *thermophilus* BioE ST107) that showed improved tolerance to acid and bile and enhanced anti-inflammatory properties compared to other potential probiotics. In order to identify the specific microbes isolated, we used 16S rRNA gene sequencing to determine the phylogenetic relationships among organisms and identify closely related species (Fig 1). We compared the 16S ribosomal DNA sequences of the isolated strains with those available in the NCBI BLAST database (100% homology). Although the *Bifidobacterium* species constitute almost 10% of the typical human intestinal microbiota, they have been isolated from the neonatal gut as the earliest and most abundant bacterial colonizers. Some *Bifidobacterium* spp. were isolated from our samples, but the commercial probiotics strains with high functionality were not selected. A relatively high percentage of *Lactobacillus*, mainly *L*. *paracasei*, *L*. *plantarum*, and *Lactobacillus acidophilus* has been previously observed in infant stool [31]. These results suggest that *Bifidobacterium* are sensitive to oxygen environment for survival than *Lactobacillus*.

We used the API 50CH fermentation system to assess the carbohydrate fermentation abilities of the isolated LAB strains (Table 2). This system supports the metabolic characterization of strains on a broad range of individual substrates with respect to enzymatic type and activity level. In this analysis, *L*. *plantarum* BioE LPL59 exhibited strong fermentation of *N*-acetyl glucosamine, amygdalin, and arbutin. In particular, *N*-acetyl glucosamine is a known component of gram-positive bacterial peptidoglycan, a major compound in the cell wall [32]. Among the genes in *L*. *plantarum* WCFS1 involved in immunomodulation are those belonging to the *N*-acetyl-glucosamine/galactosamine phosphotransferase system [33]. This suggests that *L*. *plantarum* BioE LPL59 may possess immunomodulatory genes involved in supporting cell shape or modulating surface properties. However, this ability was not detected in *L*. *paracasei* BioE LP08 or *L*. *fermentum* BioE LF11. Only *L*. *fermentum* BioE LF11 exhibited the ability to ferment d-xylitol, which can be found in many fruits, vegetables, and mushrooms [34], suggesting that *L*. *fermentum* BioE LF11 supports the reduction of d-xylitol to xylitol, similar to strains of *S*. *avium* and *L*. *casei* [35].

In order to be effective, probiotic bacteria must be able to survive travel from the upper digestive tract to the large intestine [36]. It has been previously reported that acidity has a strongly negative effect on bacterial growth and viability during passage through the stomach [37]. Bile plays an essential role in specific and non-specific defence mechanisms in the gut, and the concentration of bile salts is the primary determinant of the strength of its inhibitory effects [38]. The physiological concentrations of human bile salts range from 0.3 to 0.5% [39, 40]. Because of its similarity to human bile salts, 0.3% ox bile (Oxgall) solution is the most commonly used substitute [41, 42]. We performed an assessment of the tolerance to acid and bile salts for each strain without separation of each test. We found that although *L*. *paracasei* BioE LP08, *L*. *plantarum* BioE LPL59, and *E*. *faecalis* BioE EF71 exhibited reduced survival rates in acidic conditions, all three isolates exhibited improved tolerances to stimulated gastric juice and bile salts compared with those of *L*.*GG*. This suggests that resistant derivatives could be obtained from these strains [43] or that the strains could adapt to the presence of acid and bile salts to enhance their resistance to gastrointestinal factors that compromise probiotic survival [44].

An important feature of probiotics within the intestinal microbiota is their capacity for adhesion to the intestinal epithelium. Moreover, adherence is a factor in the competitive exclusion of enteropathogens [45] and stimulation of the immune system [46]. The Caco-2 cell line was originally isolated from a human colon adenocarcinoma [47]. In the present study, Caco-2 cells, which have been used as an *in vitro* model for the intestinal epithelium, were used to assess the adhesion abilities of the isolated strains [48, 49]. The adherence of *E*. *faecalis* BioE-EF71, *L*. *plantarum* BioE LPL59, and *S*. *thermophilus* BioE ST107 was almost twice that of *L*.*GG*.

Resistance to antibiotics is common among bacteria. The CLSI agar dilution procedure is the gold standard reference method for anaerobic antibiotic susceptibility testing [50]. Our antibiotic resistance tests indicated that the three *Lactobacillus* strains were all resistant to GMN, KMN, SMN, and VAN. These results are consistent with those of previous studies [51, 52]. Resistance to VAN is usually intrinsic, chromosomally encoded, and not transmissible [53]. The *S*. *thermophilus* strain was resistant to CIP, GMN, KMN, and TMP, similar to the results of previous studies [54]. The *E*. *faecalis* strain was resistant to CMN, ERY, GMN, KMN, SMN, TET, and TMP and susceptible to AMP, CHL, and IPM. Previous studies have reported antibiotic resistance rates of *E*. *faecalis* isolates to ERY, TET, and GMN of 82.2, 88.6, and 49.3%, respectively, and no resistance to ampicillin has been detected [55]. Among the antibiotic susceptibility studies performed in other countries, the highest resistance appears to be to TET and ERY, with resistance rates of 55–100 and 45–100%, respectively [56, 57]. Differences between species in terms of resistance to other antibiotics have also been observed.

When ingested, probiotics exert several health-promoting effects, including maintenance of the gut barrier function and modulation of the host immune system [58, 59]. It has been suggested that the safety of probiotics should be evaluated by detecting changes in immune parameters [60] due to growing evidence that probiotics, especially *Lactobacillus* and *Bifidobacterium*, have immunomodulatory properties. Macrophages sense bacteria-associated molecular patterns through Toll-like receptors (TLRs). The activation of TLR leads to a variety of signalling cascades, triggering T-cell differentiation into Helper T cells or regulatory T cells [61, 62]. Therefore, many changes of macrophage-derived cytokines could affect the immune response. In this regard, we found that *E*. *faecalis* BioE EF71, *L*. *fermentum* BioE LF11, *L*. *paracasei* BioE LP08, *L*. *plantarum* BioE LPL59, and *S*. *thermophilus* BioE ST107 exerted immunomodulatory effects when co-incubated with murine macrophages. Decreased levels of the pro-inflammatory cytokines (IL-6 and TNF-α) were observed in the supernatants macrophages treated with each strain. Surprisingly, *L*. *fermentum* BioE LF11 inhibited LPS-induced IL-6 and TNF-α production more effectively than another previously reported reference (*L*. *fermentum* KCTC 5048) [63] and also stimulated the production of IL-10 more effectively. *E*. *faecalis* BioE EF71 and *L*. *paracasei* BioE LP08 also attenuated LPS-induced TNF-α levels more effectively than other references (*E*. *faecalis* KCTC 3206 and *L*. *paracasei* KCTC 3265, respectively) [13]. In addition, increased levels of the anti-inflammatory marker IL-10 were found in the supernatants of macrophages treated with *L*. *plantarum* BioE LPL59 and *S*. *thermophilus* BioE ST107. Previous studies have reported that the strain *L*. *plantarum* CGMCC1258 results in a decrease in the transcript abundance of *TNF* [64] and that *L*. *paracasei* induces TLR-9 expression and TGF-β2 secretion [65]. Thus, we suggest that the five strains isolated in this study are likely to be recognized by a combination of receptors to regulate the immune response after inflammatory stimulus. We speculate that the use of these strains as probiotics may improve the balance between pro-inflammatory and anti-inflammatory cytokines by encountering with butyrate, which serves as a major source of energy for the colonic epithelium and has anti-inflammatory properties [66].

## Conclusions

This study screened 118 unique bacterial isolates from Korean infant stools and further characterized 20 isolates for their potential probiotic properties. Five of these, in particular *E*. *faecalis* BioE EF71, *L*. *paracasei* BioE LF11, and *L*. *plantarum* BioE LPL59, demonstrated good *in vitro* gastrointestinal tolerance. *E*. *faecalis* BioE EF71, *L*. *plantarum* BioE LPL59, and *S*. *thermophilus* BioE ST107 showed strong adherence to intestinal cells. The five strains exhibited strong effects against LPS-induced inflammatory responses in RAW264.7 cells. These findings indicate that these probiotic isolates may be useful for the treatment of acute inflammatory responses, but in our study using only RAW 264.7 cells to determine immunomodulatory properties of test products will not necessarily provide a comprehensive picture of the immunomodulatory properties of the substance under investigation [67]. It suggested that in addition to cell lines when evaluating immune bioactivity of substances, the response of primary cells can also be included *in vitro* response. It is necessary to further evaluate potential changes in the gut microbiota composition that may occur following the immunomodulatory effects of these probiotic strains in animal models.

## Supporting information

S1 Fig Adherence of strains to Caco-2 cells. Numeric values of adherence of strains to Caco-2 cells in Fig 2.
(PDF)

S2 Fig Cytokine levels. (a) IL-6, (b) TNF-α, (c) IL-10. Numeric values of cytokine levels in Fig 3.
(PDF)

S1 Dataset (XLSX)
