# Supplementary material for: Isolation and characterization of five novel probiotic strains from Korean infant and children faeces
Source: PLoS One. 2019 Oct 31;14(10):e0223913. doi: 10.1371/journal.pone.0223913 (PMC6822945; doi:10.1371/journal.pone.0223913)
Supplement: S1 Fig — Numeric values of adherence of strains to Caco-2 cells in Fig 2. (PDF) [file pone.0223913.s001.pdf]

S1 Fig. Adherence of strains to Caco-2 cells

	Mean(Log CFU/ ml)	S.D	S.E	Statistical method used	P value	samples
L.GG Initial cells	9.205	0.006			**p<0.05, ***p<0.001 versus initial cell number	3
L.GG Adhered cells	7.049	0.036				3
E. faecalis BioE EF71 Initial cells	9.139	0.044				3
E. faecalis BioE EF71 Adhered cells	8.668	0.022				3
L. fermentum BioE LF11 Initial cells	8.687	0.011				3
L. fermentum BioE LF11 Adhered cells	7.198	0.033				3
L. paracasei BioE LP08 Initial cells	9.618	0.008				3
L. paracasei BioE LP08 Adhered cells	7.942	0.020				3
L. plantarum BioE LPL59 Initial cells	8.948	0.074				3
L. plantarum BioE LPL59 Adhered cells	8.404	0.008				3
S. thermophilus BioE ST107 Initial cells	9.321	0.018				3
S. thermophilus BioE ST107 Adhered cells	7.793	0.084				3
