# Supplementary material for: Isolation and characterization of five novel probiotic strains from Korean infant and children faeces
Source: PLoS One. 2019 Oct 31;14(10):e0223913. doi: 10.1371/journal.pone.0223913 (PMC6822945; doi:10.1371/journal.pone.0223913)
Supplement: S2 Fig — Numeric values of cytokine levels in Fig 3. (PDF) [file pone.0223913.s002.pdf]

S2 Fig. Cytokine levels. (a) IL-6, (b) TNF-α, (c) IL-10.

**(a) IL-6**		Mean(pg/ml)	S.D	S.E	Statistical method used	P value	samples
Control		0.84	0.27			***p<0.001 versus negative control	3
LPS		83.10	3.42				3
LPS+	*E. faecalis* KCTC3206	1.84	0.07			###p<0.001 versus LPS-treated sample $$p<0.01, $$$p<0.001 versus KCTC registered strain of the same species	3
	E. faecalis BioE EF71	1.65	0.39				3
	L. fermentum KCTC5048	42.01	4.42				3
	L. fermentum BioE LF11	1.33	0.20				3
	L. paracasei KCTC3265	0.47	0.11				3
	L. paracasei BioE LP08	1.28	0.19				3
	L. plantarum KCTC3108	11.40	0.67				3
	L. plantarum BioE LPL59	29.82	3.84				3
	S. thermophilus KCTC3658	1.42	1.31				3
	S. thermophilus BioE ST107	11.03	5.44				3
	L. rhamnosus GG	2.47	0.20				3

**(b) TNF-α**		Mean(pg/ml)	S.D	S.E	Statistical method used	P value	samples
Control		203.43	39.87			***p<0.001 versus negative control	3
LPS		992.57	64.23				3
LPS+	*E. faecalis* KCTC3206	515.39	168.50			###p<0.001 versus LPS-treated sample $$p<0.01, $$$p<0.001 versus KCTC registered strain of the same species	3
	E. faecalis BioE EF71	296.27	37.20				3
	L. fermentum KCTC5048	111.18	7.45				3
	L. fermentum BioE LF11	8.73	3.76				3
	L. paracasei KCTC3265	118.83	27.77				3
	L. paracasei BioE LP08	3.13	3.13				3
	L. plantarum KCTC3108	148.17	37.73				3
	L. plantarum BioE LPL59	492.27	8.28				3
	S. thermophilus KCTC3658	1031.47	259.82				3
	S. thermophilus BioE ST107	884.50	263.56				3
	L. rhamnosus GG	26.23	7.34				3

**(c) IL-10**		Mean(pg/ml)	S.D	S.E	Statistical method used	P value	samples
Control		46.30	2.68			***p<0.001 versus negative control	3
LPS		47.89	5.92				3
LPS+	*E. faecalis* KCTC3206	79.88	23.13			##p<0.01, ###p<0.001 versus LPS-treated sample $$$p<0.001 versus KCTC registered strain of the same species	3
	E. faecalis BioE EF71	42.35	18.21				3
	L. fermentum KCTC5048	261.00	55.90				3
	L. fermentum BioE LF11	208.51	19.01				3
	L. paracasei KCTC3265	32.90	0.98				3
	L. paracasei BioE LP08	1.65	0.65				3
	L. plantarum KCTC3108	12.49	2.35				3
	L. plantarum BioE LPL59	254.61	29.95				3
	S. thermophilus KCTC3658	154.29	16.61				3
	S. thermophilus BioE ST107	130.34	35.78				3
	L. rhamnosus GG	8.54	1.77				3
